# Supplementary material for: Calf Circumference, a Valuable Tool to Predict Sarcopenia in Older People Hospitalized with Hip Fracture
Source: Nutrients. 2022 Oct 12;14(20):4255. doi: 10.3390/nu14204255 (PMC9608637; doi:10.3390/nu14204255)
Supplement: Supplementary file 1 [file nutrients-14-04255-s001.zip › nutrients-1970985-supplementary.pdf]

nutrients

MDPI

*Original Article*

# Calf Circumference, a Valuable Tool to Predict Sarcopenia in Older People Hospitalized with Hip Fracture

**Keith Borges [1], Reyes Artacho [1,*], Rosa Jodar-Graus [2], Esther Molina-Montes [1,3,4] and María Dolores Ruiz-López [1,3,*]**

**Supplementary table S1.** Characteristics of women patients hospitalized for hip fractures according to the presence or absence of sarcopenia (80 women older patients with hip fracture).

| | Non-sarcopenic group n=55 | Sarcopenic group n=25 | OR [95% CI] | *p* |
|---|---|---|---|---|
| **Age (years)** | 83.7 (±6.84) | 83.9 (±7.20) | 1.00 [0.94-1.08] | 0.902 |
| **Living arrangement** | | | | |
| **Other family members** | 37 (67.3%) | 18 (72.0%) | Ref. | Ref. |
| **Nursing home residents** | 7 (12.7%) | 3 (12.0%) | 0.90 [0.17-3.79] | 0.895 |
| **Alone** | 6 (10.9%) | 1 (4.00%) | 0.38 [0.01-2.59] | 0.367 |
| **Partner** | 5 (9.09%) | 3 (12.0%) | 1.25 [0.22-5.93] | 0.785 |
| **CCI** | | | | |
| High comorbidity | 54 (98.2%) | 25 (100%) | Ref. | Ref. |
| Low comorbidity | 1 (1.82%) | 0 (0.00%) | ND | |
| **Nº of medications/day** | 6.98 (±3.68) | 6.64 (±3.41) | 0.97 [0.85-1.11] | 0.691 |
| **Length of hospital stay (days)** | 11.3 (±6.90) | 11.1 (±8.53) | 1.00 [0.93-1.06] | 0.922 |
| **Weight (kg)** | 69.9 (±9.85) | 61.0 (±8.24) | 0.90 [0.85-0.96] | 0.001 |
| **Height (m)** | 1.58 (±0.06) | 1.59 (±0.05) | 1.87 [0.00-7891] | 0.884 |
| **BMI (kg/m$^2$)** | 27.9 (±4.18) | 24.2 (±3.03) | 0.76 [0.64-0.89] | 0.001 |
| **BMI categories** | | | | |
| Normal weight | 20 (36.4%) | 14 (56.0%) | Ref. | Ref. |
| Underweight | 4 (7.27%) | 7 (28.0%) | 2.42 [0.59-11.2] | 0.221 |
| Overweight | 17 (30.9%) | 3 (12.0%) | 0.27 [0.05-1.00] | 0.051 |
| Obese | 14 (25.5%) | 1 (4.00%) | 0.12 [0.00-0.71] | 0.016 |
| **CC (cm)** | 32.0 (±2.88) | 28.8 (±3.61) | 0.65 [0.51-0.83] | 0.001 |
| **CC categories** | | | | |
| Low | 15 (27.3%) | 21 (84.0%) | Ref. | Ref. |
| Normal | 40 (72.7%) | 4 (16.0%) | 0.08 [0.02-0.24] | <0.001 |
| **BI** | 60.1 (±27.6) | 53.0 (±27.2) | 0.99 [0.97-1.01] | 0.284 |
| **BI categories** | | | | |
| Independent | 2 (3.64%) | 0 (0.00%) | Ref. | Ref. |
| Mild dependent | 10 (18.2%) | 5 (20.0%) | ND | |
| Moderate dependent | 12 (21.8%) | 2 (8.00%) | ND | |
| Severe dependent | 26 (47.3%) | 17 (68.0%) | ND | |
| Total dependent | 5 (9.09%) | 1 (4.00%) | ND | |
| **MNA-SF** | 10.3 (±2.45) | 8.76 (±2.65) | 0.79 [0.65-0.96] | 0.015 |

| | | | | |
|---|---|---|---|---|
| **MNA-SF categories** | | | | |
| Normal | 19 (34.5%) | 2 (8.00%) | Ref. | Ref. |
| At risk of nutrition | 28 (50.9%) | 13 (52.0%) | 4.10 [0.96-31.1] | 0.057 |
| Malnourished | 8 (14.5%) | 10 (40.0%) | 10.6 [2.13-88.4] | 0.003 |
| **MEDAS** | 0.73 (±0.45) | 0.68 (±0.48) | 0.80 [0.28-2.23] | 0.665 |
| **MEDAS categories** | | | | |
| Low | 13 (23.6%) | 5 (20.0%) | Ref. | Ref. |
| High | 42 (76.4%) | 20 (80.0%) | 1.22 [0.39-4.33] | 0.742 |

ASMM (Appendicular Skeletal Muscle Mass); BI (Barthel Index); BMI (Body Mass Index); CC (Calf Circumference); CCI (Charlson Comorbidity Index); MEDAS (Mediterranean Diet Adherence Screener); MNA-SF (Mini Nutritional Assessment-Short Form). ND (not determined). Values are means (± SD) assuming normal distributed data or frequencies (%). OR (Odds Ratio), CI=95% (Confidence Interval) [lower CI- upper CI] and p-value (*p*).

**Supplementary table S2.** Characteristics of older women hospitalized for hip fractures according to CC (80 older women patients with hip fracture).

| | **Low CC group** *n*=36 | **Normal CC group** *n*=44 | **OR [95% CI]** | ***p*** |
|---|---|---|---|---|
| **Age (years)** | 86.1 (±7.13) | 81.9 (±6.18) | 0.91 [0.84-0.98] | 0.009 |
| **Living arrangement** | | | | |
| Other family members | 25 (69.4%) | 30 (68.2%) | Ref. | Ref. |
| Nursing home residents | 5 (13.9%) | 5 (11.4%) | 0.84 [0.20-3.44] | 0.799 |
| Alone | 2 (5.56%) | 5 (11.4%) | 1.98 [0.37-16.5] | 0.437 |
| Partner | 4 (11.1%) | 4 (9.09%) | 0.84 [0.17-4.06] | 0.819 |
| **CCI** | | | | |
| High comorbidity | 36 (100%) | 43 (97.7%) | Ref. | Ref. |
| Low comorbidity | 0 (0.00%) | 1 (2.27%) | ND | |
| **Nº of medications/day** | 6.69 (±2.92) | 7.02 (±4.08) | 1.03 [0.91-1.16] | 0.682 |
| **Length of hospital stay (days)** | 10.2 (±7.51) | 12.0 (±7.28) | 1.04 [0.97-1.11] | 0.291 |
| **Weight (kg)** | 61.9 (±8.06) | 71.3 (±9.88) | 1.12 [1.06-1.19] | <0.001 |
| **Height (m)** | 1.58 (±0.05) | 1.59 (±0.06) | 226 [0.07-701016] | 0.186 |
| **BMI (kg/m²)** | 24.9 (±3.01) | 28.2 (±4.48) | 1.26 [1.10-1.45] | 0.001 |
| **BMI categories** | | | | |
| Normal weight | 22 (61.1%) | 12 (27.3%) | Ref. | Ref. |
| Underweight | 7 (19.4%) | 4 (9.09%) | 1.06 [0.23-4.40] | 0.940 |
| Overweight | 5 (13.9%) | 15 (34.1%) | 5.23 [1.58-20.0] | 0.006 |
| Obese | 2 (5.56%) | 13 (29.5%) | 10.7 [2.40-84.5] | 0.001 |
| **BI** | 56.4 (±27.6) | 59.1 (±27.6) | 1.00 [0.99-1.02] | 0.660 |
| **BI categories** | | | | |
| Independent | 1 (2.78%) | 1 (2.27%) | Ref. | Ref. |
| Mild dependent | 7 (19.4%) | 8 (18.2%) | 1.13 [0.03-49.9] | 0.941 |
| Moderate dependent | 3 (8.33%) | 11 (25.0%) | 3.32 [0.07-154] | 0.500 |

| | | | | |
|---|---|---|---|---|
| Severe dependent | 22 (61.1%) | 21 (47.7%) | 0.96 [0.02-38.9] | 0.978 |
| Total dependent | 3 (8.33%) | 3 (6.82%) | 1.00 [0.02-51.4] | 1.000 |
| **MNA-SF** | 8.86 (±2.37) | 10.6 (±2.53) | 1.34 [1.10-1.63] | 0.004 |
| **MNA-SF categories** | | | | |
| Normal | 3 (8.33%) | 18 (40.9%) | Ref. | Ref. |
| At risk of nutrition | 21 (58.3%) | 20 (45.5%) | 0.17 [0.03-0.60] | 0.005 |
| Malnourished | 12 (33.3%) | 6 (13.6%) | 0.09 [0.02-0.41] | 0.001 |
| **MEDAS** | 0.78 (±0.42) | 0.66 (±0.48) | 0.55 [0.20-1.51] | 0.246 |
| **MEDAS categories** | | | | |
| Low | 6 (16.7%) | 12 (27.3%) | Ref. | Ref. |
| High | 30 (83.3%) | 32 (72.7%) | 0.54 [0.17-1.61] | 0.275 |
| **Grip Strength (kg)** | 9.96 (±3.87) | 11.5 (±4.43) | 1.09 [0.98-1.22] | 0.110 |
| **Grip categories** | * | | | |
| Low | 35 (97.2%) | 39 (88.6%) | Ref. | Ref. |
| Normal | 1 (2.78%) | 5 (11.4%) | 4.00 [0.58-110] | 0.177 |
| **ASMM/height$^2$ (kg/m$^2$)** | 6.12 (±1.29) | 7.21 (±1.16) | 2.18 [1.38-3.42] | 0.001 |
| **ASMM/height$^2$ categories** | | | | |
| Low | 22 (61.1%) | 5 (11.4%) | Ref. | Ref. |
| Normal | 14 (38.9%) | 39 (88.6%) | 11.6 [3.89-41.0] | <0.001 |
| **Sarcopenia diagnosis** | | | | |
| Non-sarcopenic | 15 (41.7%) | 40 (90.9%) | Ref. | Ref. |
| Sarcopenic | 21 (58.3%) | 4 (9.09%) | 0.08 [0.02-0.24] | <0.001 |

ASMM (Appendicular Skeletal Muscle Mass); BI (Barthel Index); BMI (Body Mass Index); CC (Calf Circumference); CCI (Charlson Comorbidity Index); MEDAS (Mediterranean Diet Adherence Screener); MNA-SF (Mini Nutritional Assessment-Short Form). ND (not determined). Values are means (± SD) assuming normal distributed data or frequencies (%). OR (Odds Ratio), CI=95% (Confidence Interval) [lower CI- upper CI] and p-value (*p*).
